# Sp1 S-Sulfhydration Induced by Hydrogen Sulfide Inhibits Inflammation via HDAC6/MyD88/NF-κB Signaling Pathway in Adjuvant-Induced Arthritis

**DOI:** 10.3390/antiox11040732

**Published:** 2022-04-07

**Authors:** Meng Li, Wei Hu, Ran Wang, Zhaoyi Li, Yue Yu, Yue Zhuo, Yida Zhang, Zhou Wang, Yuanye Qiu, Keyuan Chen, Qian Ding, Wei Qi, Menglin Zhu, Yizhun Zhu

**Affiliations:** 1 State Key Laboratory of Quality Research in Chinese Medicine, Faculty of Chinese Medicine, Macau University of Science and Technology, Macau 999078, China; mengli@gwcmc.org (M.L.); 1909853xct30001@student.must.edu.mo (W.H.); 1809853pct30001@student.must.edu.mo (R.W.); 1909853gct20008@student.must.edu.mo (Z.L.); 1709853fct30001@student.must.edu.mo (Y.Y.); zhuoyue@gzucm.edu.cn (Y.Z.); 1609853ect30002@student.must.edu.mo (Y.Z.); 1709853jct30002@student.must.edu.mo (Z.W.); 17098533ct30001@student.must.edu.mo (Y.Q.); 16098539ct30001@student.must.edu.mo (K.C.); 1909853qct30001@student.must.edu.mo (Q.D.); wqi@must.edu.mo (W.Q.); 2009853ect30003@student.must.edu.mo (M.Z.); 2 Department of Pediatric Surgery, Guangzhou Institute of Pediatrics, Guangdong Provincial Key Laboratory of Research in Structural Birth Defect Disease, Guangzhou Women and Children’s Medical Center, Guangzhou Medical University, Guangzhou 510623, China; 3 Science and Technology Innovation Center, Guangzhou University of Chinese Medicine, Guangzhou 510006, China; 4 State Key Laboratory of Respiratory Disease, National Clinical Center for Respiratory Diseases, Guangzhou Institute of Respiratory Diseases, First Affiliated Hospital, Guangzhou Medical University, Guangzhou 510120, China; 5 School of Pharmacy, Macau University of Science and Technology, Macau 999078, China; 6 Shanghai Key Laboratory of Bioactive Small Molecules, Department of Pharmacology, School of Pharmacy, Fudan University, Shanghai 201203, China

**Keywords:** HDAC6, Sp1, H_2_S, rheumatoid arthritis

## Abstract

Histone deacetylase 6 (HDAC6) acts as a regulator of the nuclear factor kappa-B (NF-κB) signaling pathway by deacetylating the non-histone protein myeloid differentiation primary response 88 (MyD88) at lysine residues, which is an adapter protein for the Toll-like receptor (TLR) and interleukin (IL)-1β receptor. Over-activated immune responses, induced by infiltrated immune cells, excessively trigger the NF-κB signaling pathway in other effector cells and contribute to the development of rheumatoid arthritis (RA). It has also been reported that HDAC6 can promote the activation of the NF-κB signaling pathway. In the present study, we showed that HDAC6 protein level was increased in the synovium tissues of adjuvant-induced arthritis rats. In addition, hydrogen sulfide (H_2_S) donor S-propargyl-cysteine (SPRC) can inhibit HDAC6 expression and alleviate inflammatory response in vivo. In vitro study revealed that HDAC6 overexpression activated the NF-κB signaling pathway by deacetylating MyD88. Meanwhile, sodium hydrosulfide (NaHS) or HDAC6 inhibitor tubastatin A (tubA) suppressed the pro-inflammatory function of HDAC6. Furthermore, the reduced expression of HDAC6 appeared to result from transcriptional inhibition by S-sulfhydrating specificity protein 1 (Sp1), which is a transcription factor of HDAC6. Our results demonstrate that Sp1 can regulate HDAC6 expression, and S-sulfhydration of Sp1 by antioxidant molecular H_2_S ameliorates RA progression via the HDAC6/MyD88/NF-κB signaling pathway.

## 1. Introduction

Rheumatoid arthritis (RA) is a worldwide autoimmune disease characterized by progressive disability, systemic complications, and early death [1]. In the development of RA, the synovium tissue undergoes a striking transformation induced by the infiltration of immune and inflammatory cells, as well as the hyperplasia of synovial lining [2]. Fibroblast-like synoviocytes (FLS) and macrophage-like synoviocytes (MLS), which compose the synovial intimal lining, are excessively activated in RA synovium tissues. The main cytokines secreted by MLS are the granulocyte-macrophage colony-stimulating factor (GM-CSF), tumor necrosis factor-α (TNF-α), and interleukin-1β (IL-1β), which can further stimulate FLS to secrete IL-6, matrix metalloproteinases (MMPs), collagenases, macrophage colony-stimulating factor (M-CSF), and the receptor activator of nuclear factor kappa-B ligand (RANKL) [3,4]. The interaction of these two cellular components (MLS/FLS) in RA synovium is vital for the initiation and maintenance of inflammation and subsequent damage to the joints. It is well acknowledged that FLS are directly responsible for cartilage destruction, and can also drive both inflammation and autoimmunity [5]. Meanwhile, FLS integrate many stimuli that promote disease, amplifying inflammation and tissue damage in the synovium [6]. In the last decade, anti-TNF-α monoclonal antibodies (infliximab, etanercept, and adalimumab) have been used successfully in the treatment of RA [7]. However, this kind of therapy is accompanied by several side-effects, such as infections, malignancies, heart failure, and so on, which need more attention in clinical application [8,9]. Hence, exploring new strategies for RA therapy is ongoing and imperative.

Epigenetic modifications include DNA methylation and histone post-translational alterations (methylation, acetylation, ubiquitination, or phosphorylation), both of which control cell-specific gene expression patterns [10,11]. However, with the exploration and development of epigenetics, the epigenetic enzyme histone deacetylase 6 (HDAC6) attracts increasing attention in RA study as it deacetylates not only histones, but also non-histone proteins including α-tubulin and myeloid differentiation primary response 88 (MyD88) [12,13]. Moreover, HDAC6 was identified as a pro-inflammatory epigenetic enzyme, which is closely related to its deacetylation function on MyD88 [14]. MyD88 is an essential adaptor molecule for the Toll-like receptor (TLR) and IL-1 receptors in the NF-κB signaling pathway, which can be deacetylated by HDAC6. HDAC6 belongs to class II HDAC (HDAC IIa 4, 5, 7, 9, and IIb 6, 10). Class I (HDAC1, 2, 3, 8), II, and IV (HDAC11) HDACs are Zn^2+^-dependent enzymes, whereas class III HDAC (Sirtuin1–7) require NAD+ for their enzymatic activity [15]. HDAC6 has been identified to be involved in multiple cellular processes, including cell migration, intracellular transport, and neurodegenerative disorders [12,16,17,18]. Recent studies also revealed the vital roles of HDAC6 in the innate immune response and inflammation by regulating the nuclear factor kappa-B (NF-κB) signaling pathway [19,20]. Moreover, inhibition of HDAC6 demonstrated anti-inflammatory and anti-rheumatic effects in collagen-induced arthritis (CIA) [21,22]. Therefore, HDAC6 could be a potential target for the effective therapy of RA.

Hydrogen sulfide (H_2_S) has been proposed as the third endogenous gasotransmitter, following nitric oxide (NO) and carbon monoxide (CO), which can penetrate the cellular membrane without the requirement of any specific transporter [23]. Endogenous H_2_S is generated from cysteine in the reaction catalyzed by three main enzymes: cystathionine β-synthase (CBS), cystathionine γ-lyase (CSE or CTH), and 3-mercaptopyruvate sulfurtransferase (3-MST or MPST) [24]. H_2_S has widely been demonstrated to possess cardio-protective and pro-angiogenic effects in vivo and in vitro [25,26], as well as inhibitory effects on platelet aggregation, cytoprotective effect, and anti-apoptotic activity [27,28,29]. However, the anti-inflammation property of H_2_S is the most frequently reported activity [30,31,32]. Several studies have confirmed that H_2_S inhibits the expression of NF-κB-regulated pro-inflammatory cytokines, such as TNF-α, IL-1β, IL-6, cyclooxygenase-2 (COX2), and inducible nitric oxide synthase (iNOS) in macrophages, cardiac cell lines, and chondrocytes [33,34,35]. It was revealed that H_2_S dependently S-sulfhydrates Cys38 of p65 to retain NF-κB in the cytosol and inhibit its DNA binding activity, which leads to reduced inflammation [36]. In addition, S-sulfhydrates of p65 inhibits the over-production of inflammation cytokines and reactive oxygen species (ROS) [37]. In the reaction of S-sulfhydration, sulfane sulfur is transferred to the –SH groups, forming hydropersulfides, which is a cellular redox regulation process [38,39]. H_2_S-induced S-sulfhydration can inhibit the production of mitochondrial reactive oxygen species (ROS) by targeting the Cys-59 residue of p66Shc, which prevents its phosphorylation [40]. Therefore, there is a close connection between S-sulfhydration, inflammation, and antioxidation.

A previous study evidenced that S-propargyl-cysteine (SPRC), an endogenous H_2_S donor, depressed inflammatory mediators in IL-1β-stimulated FLS and adjuvant-induced arthritis (AIA) rats through the modulation of the nuclear factor E2-related factor 2 (Nrf2)/heme oxygenase-1 (HO-1) signaling pathway [41]. Thus, we hypothesized that SPRC/H_2_S might exert an anti-RA effect by targeting HDAC6 and consequently inhibiting NF-κB signaling. This study was designed to verify this hypothesis and increase the potential of H_2_S in RA therapy.

## 2. Materials and Methods

### 2.1. Materials

Dulbecco’s modified Eagle’s medium (DMEM), fetal bovine serum (FBS), 100× Penicillin Streptomycin Glutamine (PSG, 10,000 Units/mL penicillin, 10,000 μg/mL streptomycin, and 29.2 mg/mL L-glutamine), trypsin-EDTA, Opti-MEM, and phosphate buffer saline (PBS) were purchased from Life Technologies (Gaithersburg, MD, USA). Lipofectamine 2000 and lipofectamine RNAiMAX reagent were obtained from Thermo Fisher Scientific (Waltham, MA, USA). Tubastatin A (tubA) was purchased from MedChemExpress (Monmouth Junction, NJ, USA). Dimethyl sulfoxide (DMSO), NaHS, Na_2_S, lipopolysaccharide (LPS), monobromobimane (MBB), and mineral oil were purchased from Sigma-Aldrich (St. Louis, MO, USA). Methotrexate (MTX) hydrate was provided by J&K Scientific (Guangzhou, Guangdong, China). Biotin-HPDP was obtained from Cayman Chemical (Ann Arbor, MI, USA). Desiccated *M. tuberculosis* H37 Ra was obtained from BD Difco (Sparks, MD, USA). TRIzol reagent was provided by Invitrogen (Carlsbad, CA, USA), and iScript cDNA synthesis kit was purchased from Bio-Rad (Hercules, CA, USA). FastStar Universal SYBR Green Master (ROX) was obtained from Roche Diagnostics (Indianapolis, IN, USA). Radio-immunoprecipitation assay (RIPA) buffer was obtained from Cell Signaling Technology (Danvers, MA, USA). Primary antibodies against HDAC6, MyD88, IκBα, p-IκBα (Ser32/36), p65, p-p65 (Ser536), and acetylated lysine were purchased from Cell Signaling Technology. Protein A/G PLUS-Agarose was provided by Santa Cruz Biotechnology (Santa Cruz, CA, USA). Primary antibodies against CSE and acetylated α-tubulin were obtained from Santa Cruz Biotechnology. The primary antibody against α-tubulin was provided by HuaAn Biotechnology (Hangzhou, Zhejiang, China). Streptavidin agarose and the primary antibody against Sp1 were purchased from Millipore (Bedford, MA, USA). The primary antibody against β-actin was obtained from Proteintech Group (Wuhan, Hubei, China). Peroxidase-conjugated AffiniPure goat anti-mouse and -rabbit IgG (H + L) antibodies were purchased from Jackson Laboratory (West Grove, PA, USA). The mouse anti-rabbit IgG (conformation specific) antibody (HRP conjugate) was obtained from Cell Signaling Technology. A chromatin immunoprecipitation (ChIP) kit was provided by BersinBio (Guangzhou, Guangdong, China). A dual-luciferase reporter assay kit was purchased from BPS Bioscience (San Diego, CA, USA). All plasmids were provided by GeneChem (Shanghai, China).

### 2.2. Cells and Culture

Rat RA fibroblast-like synoviocytes (rRAFLS) were isolated from the knee joint synovium of AIA rats and maintained in DMEM supplemented with 15% FBS and 1× PSG in a humid incubator (37 °C and 5% CO_2_). rRAFLS were trypsinized with 0.05% trypsin-EDTA for subculture or experiments.

### 2.3. Animals

Male Sprague Dawley (SD) rats (4–5 weeks old, 120–140 g) were purchased from the Laboratory Animal Services Center of the Chinese University of Hong Kong (Hong Kong, China). The animals were acclimated for 1 week prior to the experiment, and 200 ± 20 g rats were chosen as experimental subjects. All rats were housed under standard laboratory conditions and maintained on a 12-h light 12-h dark cycle with food and water provided ad libitum. All surgeries were performed under pentobarbital (2%, dissolved in saline, 3 mL/kg) anesthesia to minimize suffering, in accordance with the guidelines for the care and use of laboratory animals. Rat blood was collected from the aortaventralis after deep anesthesia, and euthanasia was performed by using CO_2_ in a closed container. All animal studies described herein were approved by the Animal Care and Use Committee of the Municipal Affairs Bureau of Macau (approval number AL010/DICV/SIS/2018).

### 2.4. AIA Rat Model

Rats were immunized on day 1 by a subcutaneous injection of *M. tuberculosis* suspended in mineral oil (4 mg/mL, 50 μL per rat) at the base of the tail. Rats were divided into the following 6 groups: Sham group (subcutaneously injected with saline once and treated with PBS daily, intragastric), Model group (immunized and treated with PBS daily, intragastric), MTX group (immunized and treated with MTX twice a week, 1 mg/kg, intraperitoneal), SPRC groups (immunized and treated with SPRC daily, 25, 50, or 100 mg/kg, intragastric). MTX and SPRC were dissolved in PBS. On day 30, all the rats were sacrificed. The severity of the arthritis was scored every 5 days after day 10. Inflammation of paws were graded from 0 to 4, as follows: grade 0, paws with no swelling and focal redness; grade 1, paws with swelling of finger joints; grade 2, paws with mild swelling of the ankle or wrist joints; grade 3, paws with severe inflammation of the entire paws; grade 4, paws with deformity or ankylosis. Volumes of hind footpads were determined by plethysmometer (Ugo Basile, Varese, Italy).

### 2.5. Histopathology

Rat ankle joints for histological analysis were removed and immediately fixed in 4% paraformaldehyde. Fixed samples were decalcified in EDTA and embedded in paraffin. Joint tissues were sectioned at 7 μm thickness, dewaxed using xylene, dehydrated through a gradient of alcohol, and stained with hematoxylin and eosin (H&E). The stained sections were evaluated to assess joint inflammation, including inflammatory cell infiltration, synovial congestion, fibrous tissue hyperplasia, and bone erosion.

### 2.6. H_2_S Measurement

At day 30, rat plasma samples were collected and prepared according to the method of [42,43]. Briefly, 30 μL of sample was added to 70 μL of 100 mM Tris-HCl buffer (pH 9.5, 0.1 mM diethylenetriaminepentaacetic acid), followed by addition of 50 μL of 10 mM MBB. The reaction was stopped by adding 50 μL of 200 mM 5-sulfosalicylic acid after 30 min incubation at room temperature (RT). The mixture was then centrifuged (4 °C, 14,000× *g*, 10 min). 30 μL of the supernatant was added to 267 μL of 50% acetonitrile, then 3 μL of hydrocortisone internal standard solution (1.0 mg/mL) was added. Different concentrations of Na_2_S solution were prepared to produce a standard curve. All samples were injected into the Agilent 6490 Triple Quadrupole LC/MS instrument (Agilent Technologies, Palo Alto, CA, USA).

### 2.7. RNA Extraction and Quantitative Real-Time PCR (qRT-PCR)

Total RNA was extracted from cells or synovium tissues using TRIzol reagent, quantified by a NanoDrop 2000 spectrophotometer (Thermo Fisher Scientific, USA), and stored at −80 °C. One microgram of RNA was reverse transcribed using an iScript cDNA synthesis kit following the manufacturer’s recommendations. Transcripts were quantified using FastStar Universal SYBR Green Master (ROX) with the LightCycler 480 instrument (Roche Diagnostics, USA). Relative mRNA expression levels of target genes were standardized with the housekeeping gene β-actin, the value of which was set as 1. The fold change of mRNA was calculated using the 2^−ΔΔCt^ method. The rat primer sequences used in the study were as follows:

CSE-forward: 5′-TGTTGTCATGGGCTTAGTG-3′
CSE-reverse: 5′-CCATCCCATT CCTGAAGTG-3′
HDAC6-forward: 5′-GTCTCATCCTACCTGCTC-3′
HDAC6-reverse: 5′-GGCAGATGTAGATGGACT-3′
COX2-forward: 5′-TGAACACGGACTTGCTCACTTTG-3′
COX2-reverse: 5′-AGGCCTTTGCCACTGCTTGTA-3′
IL-6-forward: 5′-GACTTCACAGAGGATACC-3′
IL-6-reverse: 5′-TAAGTTGTTCTTCACAAACTCC-3′
IL-1β-forward: 5′-AAAAATGCCTCGTGCTGTCT-3′
IL-1β-reverse: 5′-TCGTTGCTTGTCTCTCCTTG-3′
RANKL-forward: 5′-CCGAGACTACGGCAAGTACC-3′
RANKL-reverse: 5′-CTGCGCTCGAAAGTACAGGA-3′
OPG-forward: 5′-GTTCTTGCACAGCTTCACCA-3′
OPG-reverse: 5′-AAACAGCCCAGTGACCATTC-3′
MMP9-forward: 5′-GGGCATCTGGGGATTGAACTCAGC-3′
MMP9-reverse: 5′-AGCGCCCGACGCACAGTAAG-3′
MMP13-forward: 5′-TGATGATGAAACCTGGACAAGCA-3′
MMP13-reverse: 5′-GAACGTCATCATCTGGGAGCA-3′
β-actin-forward: 5′-TGACAGGATGCAGAAGGAGA-3′
β-actin-reverse: 5′-TAGAGCCACCAATCCACACA-3′.

### 2.8. ChIP Assay

The binding of Sp1 with HDAC6 promoter was determined by ChIP assay, as previously described [44]. Briefly, after different treatments, rRAFLS in 100 mm dishes were fixed directly by adding formaldehyde (1%) for 10 min at 37 °C to cross-link proteins to DNA. The cells were then collected and sonicated to shear DNA to lengths between 200 bp and 600 bp. The sonicated supernatant was incubated with an antibody against Sp1 overnight at 4 °C with rotation. The samples incubated with nonspecific IgG antibody acted as the negative control. A fraction of the cross-linked protein–DNA was not precipitated, but set aside for the total chromatin examination (termed input). The chromatin–antibody complexes and the input were eluted, reversed, and purified. The aimed sequence containing the potential Sp1 binding site in the HDAC6 promoter was amplified by PCR using respective primers. The sequences of the primers used were as follows:

HDAC6-forward: 5′-ACAACCCACAGGCTGAGAAG-3′
HDAC6-reverse: 5′-GTCACCATGCAGAGCAGAGT-3′
GAPDH-forward: 5′-TGAGAGAGGCCCAGCTACTC-3′
GAPDH-reverse: 5′-AGGGCTGCAGTCCGTATTTA-3′.

The following PCR program was used: 94 °C for 5 min, followed by 39 cycles of 95 °C for 20 s, 55 °C for 30 s, and 72 °C for 30 s, followed by a final extension at 72 °C for 15 min. The PCR products were electrophoresed on a 2% agarose gel. The input was used as a positive control. Quantitative analysis was determined by qRT-PCR, and binding intensity of Sp1 with the promoters was normalized to the level of input using the same primers.

### 2.9. Biotin Switch Assay

Protein S-sulfhydration was performed and modified as described previously [45]. Briefly, rRAFLS were disrupted via ultrasonic treatment in HEN buffer (250 mM Hepes-NaOH (pH 7.7), 1 mM EDTA, and 0.1 mM neocuproine) supplemented with 100 µM deferoxamine, and centrifuged at 14,000× *g* for 10 min at 4 °C. Cell lysates were added to blocking buffer (HEN buffer with 2.5% SDS and 20 mM methyl methanethiosulfonate (MMTS)) at 50 °C for 20 min with frequent vortexing. MMTS was then removed by pre-cold acetone, and the proteins were precipitated at −20 °C for 20 min. The mixture was centrifuged at 5000× *g* for 10 min at 4 °C. After acetone removal, the proteins were resuspended in HENS buffer (HEN buffer with 1% SDS). Subsequently, 4 mM biotin-HPDP in DMSO without ascorbic acid was added to the suspension. After incubation for 3 h at RT, proteins were precipitated at −20 °C for 20 min using pre-cold acetone to remove biotin-HPDP. The mixture was centrifuged at 5000× *g* for 10 min at 4 °C. The precipitate was resuspended in neutralization buffer (20 mM Hepes-NaOH (pH7.7), 1 mM EDTA, 100 mM NaCl, and 0.5% SDS). Biotinylated proteins were precipitated using streptavidin–agarose beads after 1 h incubation at RT, which were then washed with neutralization buffer 5 times. Biotinylated proteins were eluted by 1× SDS-PAGE loading buffer and subjected to Western blotting analysis.

### 2.10. Immunoprecipitation (IP)

Cells were lysed in RIPA buffer containing a protease and phosphatase inhibitor cocktail (Roche) and then centrifuged at 14,000× *g* for 10 min at 4 °C. The supernatant was first incubated a on rotating device overnight with anti-MyD88 antibody at 4 °C. Then, protein A/G–agarose beads were added and allowed to incubate for 3 h at 4 °C on a rotating device. Subsequently, the beads were collected by centrifugation at 1000× *g* for 2 min at 4 °C, and washed five times with PBS. The immunopurified protein was eluted by boiling the beads for 5 min in 1× SDS-PAGE loading buffer. Subsequently, the supernatant was collected and analyzed by Western blotting.

### 2.11. Western Blotting

Proteins were extracted with RIPA lysis buffer containing a protease and phosphatase inhibitor cocktail (Roche, USA). The total proteins were quantified with a BCA protein assay kit (Thermo Fisher Scientific, USA) and loaded onto 8% SDS-PAGE gel. Western blotting was performed by transferring the proteins onto nitrocellulose (NC) membranes (Pall Corporation, Port Washington, New York, NY, USA) using a TransBlot system (Bio-Rad, USA). The membranes were washed in Tris-buffered saline supplemented with 0.1% Tween 20 (TBST) and then blocked with 5% skimmed milk in TBST for 1 h at RT. Afterwards, the membranes were incubated overnight at 4 °C with antibodies at the indicated dilutions, followed by incubation with HRP-conjugated secondary antibodies for 1 h at RT. Blots were visualized with UltraSignal ECL chemiluminescent substrate (4A Biotech, Beijing, China) and captured using Amersham Imager 600 (GE, Boston, MA, USA). Quantitative data were acquired using ImageJ software (NIH, Bethesda, Rockville, MD, USA).

### 2.12. Cell Transfection

The rRAFLS cells were inoculated in 60 mm dishes (5 × 105 cells per dish). For small interfering RNA (siRNA) transfection, CSE-specific siRNA (si-CSE) or scrambled siRNA (Scr) was mixed with lipofectamine RNAiMAX according to the manufacturer’s instructions. For plasmid transfection, HDAC6 overexpression (OE-HDAC6) plasmid or vector (Vec) was mixed with lipofectamine 2000. After transfection for 6 h, cells were exposed to the indicated NaHS/tubA treatments for another 6 h, when total RNA were required, or for 24 h, when proteins were required.

### 2.13. Dual-Luciferase (Firefly–Renilla) Assay

The sequence of HDAC6 promoter and the corresponding mutant were cloned and inserted into the GV354 plasmid. In 6-well plates, rRAFLS were cultured to approximately 70% confluence and then co-transfected with either wild type (WT) or mutant (Mut) HDAC6 promoter plasmid (2 μg) and Sp1 overexpression (OE-Sp1) plasmid (2 μg). After 12 h, luciferase activity was measured and normalized to the activity of Renilla luciferase.

### 2.14. Statistical Analysis

Quantitative data were presented as mean ± SEM (standard error of the mean). The calculations were performed with GraphPad Prism 7.04 software (GraphPad Software Inc., San Diego, CA, USA). Comparisons between 2 groups were analyzed by two-tailed unpaired Student’s t-tests, and among 3 or more groups were assessed by one-way analysis of variance (ANOVA) with Tukey’s post hoc multiple comparison. Values of *p* < 0.05 indicated statistical significance.

## 3. Results

### 3.1. H_2_S Inhibits the Expression of HDAC6 Gene and HDAC6-Induced Inflammatory Genes in rRAFLS

**Figure 1:**
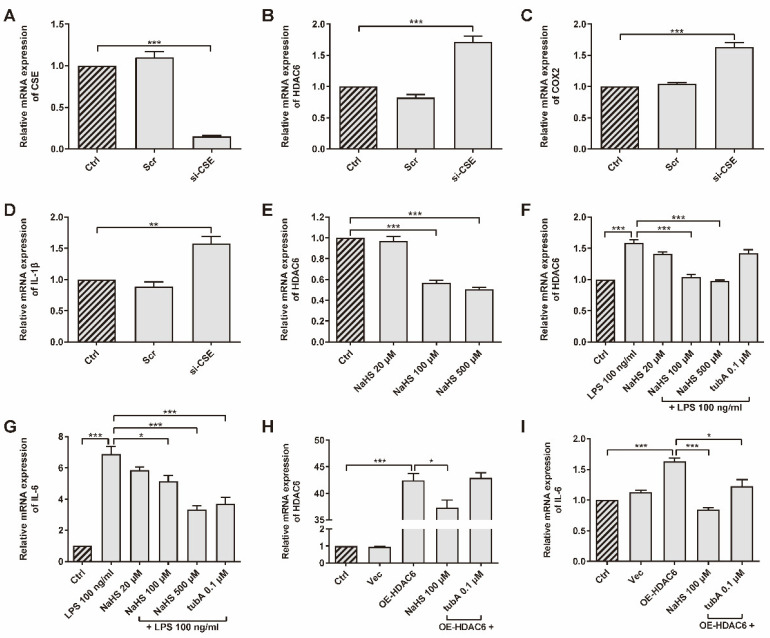
HDAC6 is a H_2_S-related pro-inflammatory factor in rRAFLS. The gene expression of (**A**) CSE, (**B**) HDAC6, (**C**) COX2, and (**D**) IL-1β in rRAFLS transfected with scrambled (Scr) or targeting CSE (si-CSE) siRNA for 6 h. (**E**) Cells in control (Crtl) group and NaHS (20, 100, 500 μM) groups were collected after 6 h treatment and then subjected to qRT-PCR. (**F**,**G**) Cells were pre-treated with NaHS (20, 100, 500 μM) and tubA (0.1 μM) for 1 h and then stimulated with LPS (100 ng/mL) for another 5 h. Then, the cells were subjected to qRT-PCR. (**H**,**I**) Cells were transfected with vector (Vec) or HDAC6 overexpression plasmid for 6 h and treated with NaHS (100 μM) and tubA (0.1 μM) for another 6 h. Then, the cells were subjected to qRT-PCR. Bars indicate the mean ± SEM. * *p* < 0.05, ** *p* < 0.01, *** *p* < 0.001. *n* = 3–4 in each group.

CSE was up-regulated in the AIA rat model after SPRC treatment, according to a previous study [41]. The association between CSE/H_2_S and HDAC6 has not yet been verified. We chose primary rRAFLS as our experiment subject upon which to perform assays. To investigate whether CSE inhibits HDAC6 expression, rRAFLS were transfected with small interfering RNA (siRNA) to silence CSE gene expression (Figure 1A). We found that CSE silencing promoted HDAC6, cyclooxygenase 2 (COX2), and IL-1β gene expression (Figure 1B–D), which suggested that CSE inhibition induced inflammation. Meanwhile, once CSE expression was inhibited, the HDAC6 protein level increased (Figure S1). The addition of exogenous H_2_S donor NaHS down-regulated HDAC6 gene expression in a dose-dependent manner (Figure 1E). Interestingly, LPS increased HDAC6 mRNA level, and NaHS blocked the change (Figure 1F). Accumulating evidence shows that LPS can activate NF-κB by targeting Toll-like receptor 4 (TLR4) [46]. Therefore, HDAC6 gene expression might be enhanced by NF-κB signaling. It is not surprising that HDAC6-specific protein inhibitor tubA did not affect HDAC6 gene expression. However, tubA reduced the IL-6 gene level, which showed a similar function as NaHS after LPS stimulation (Figure 1G), indicating that HDAC6 plays a role in LPS-induced IL-6 production. To investigate whether HDAC6 overexpression promotes inflammation responses, rRAFLS were transfected with the OE-HDAC6 plasmid to elevate HDAC6 gene level. The results exhibited that NaHS decreased HDAC6 gene expression after transfection (Figure 1H) and NaHS or tubA inhibited IL-6 gene expression induced by HDAC6 up-regulation (Figure 1I). These data demonstrate that H_2_S can regulate HDAC6 expression and inhibit LPS- or HDAC6-induced inflammation at the gene level.

### 3.2. H_2_S Donor SPRC Suppresses RA Progress in Rat AIA Model

**Figure 2:**
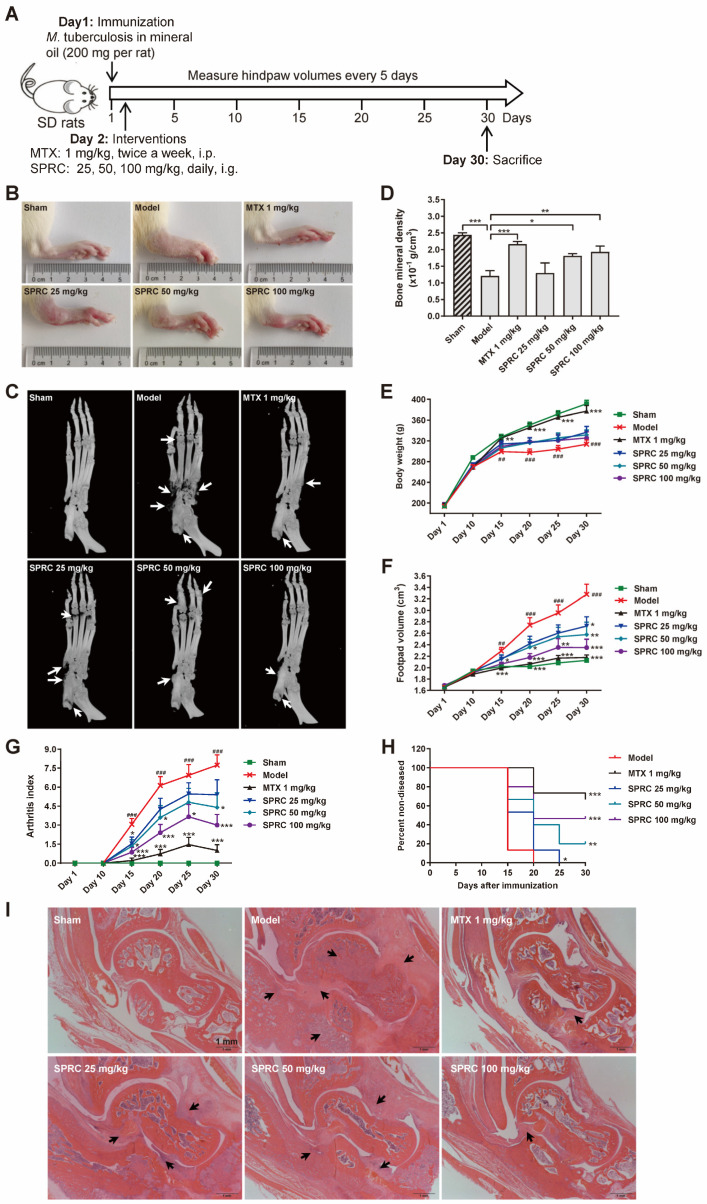
SPRC ameliorates RA symptoms in AIA rat model. (**A**) The schematic representation of in vivo experiment. (**B**) Representative right hind paws in each group. (**C**) Right hind paws were fixed in 4% paraformaldehyde and subjected to animal micro-computed tomography (micro-CT). Bone erosion parts are indicated by white arrows. (**D**) Bone mineral density of the heels was analyzed by micro-CT. (**E**) The body weight, (**F**) hind paw volume, (**G**) arthritis score, and (**H**) disease incidence were statistically analyzed. (**I**) Ankles of right hind paws were stained with hematoxylin and eosin. Typical soft tissue hyperplasia and bone destruction are indicated by black arrows (bar, 1 mm; magnification, 40×). Bars indicate the mean ± SEM. * *p* < 0.05, ** *p* < 0.01, *** *p* < 0.001. In (**C**), *n* = 5 in each group. In (**D**–**F**), *n* = 15 in each group.

To investigate the correlation between SPRC/H_2_S and HDAC6 in vivo, SD rats were immunized with heat-killed *M. tuberculosis* to establish the AIA model [47]. A schematic representation of the in vivo experiment is exhibited in Figure 2A. It was observed that SPRC ameliorated foot pad swelling in a dose-dependent manner (Figure 2B,F). Although SPRC induced a slight increase in body weight compared to the model group, there was no significance between these groups (Figure 2E). Consistently, the arthritic index from each group statistically demonstrated that SPRC inhibited joint inflammation (Figure 2G). As a transcription factor, NF-κB can promote RANKL expression to activate the RANK/RANKL/OPG signaling pathway in osteoclasts, leading to bone destruction [48]. Therefore, inhibition of inflammation contributes to bone protection. As shown in Figure 2C,D, bone erosion was suppressed after SPRC treatment, which verified our hypothesis. In addition, SPRC markedly increased the percentage of healthy rats after immunization (Figure 2H), suggesting that SPRC delayed RA progress. Alternatively, hematoxylin and eosin (H&E) staining of ankles showed obvious inhibition of synovial congestion, fibrous tissue hyperplasia, and bone erosion after SPRC treatment (Figure 2I). Expectedly, the clinical drug MTX showed a desirable anti-RA effect in the animal model.

**Figure 3:**
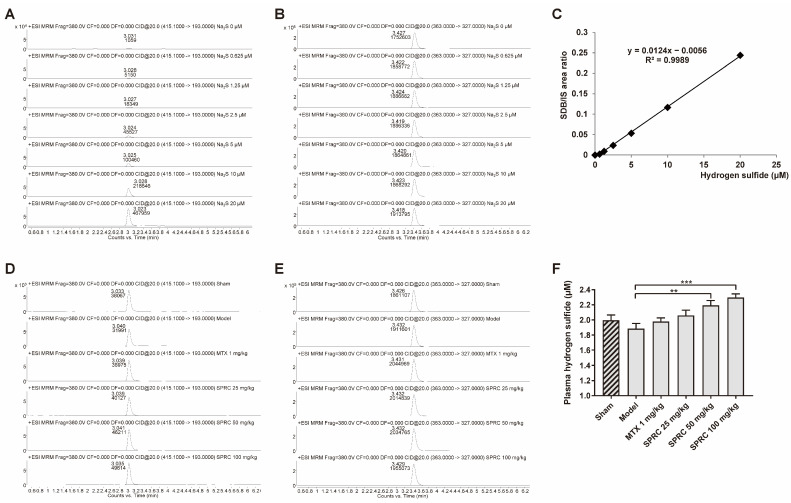
SPRC increases H_2_S level in AIA rat model. Rat plasma samples were collected and subjected to HPLC-MS for total hydrogen sulfide (S^2−^/HS^−^/H_2_S) detection. Representative peak area of derivative product SDB in (**A**) Na_2_S (0–20 μM) groups or (**D**) experimental groups were calculated by Agilent software, as well as (**B**,**E**) that of hydrocortisone. (**C**) The area ratio of SDB/IS was used to construct a standard curve. (**F**) The exact concentrations of total hydrogen sulfide in plasma were calculated using the standard curve. Bars indicate the mean ± SEM. ** *p* < 0.01, *** *p* < 0.001. *n* = 3 or 5 in each group.

Hence, to further study whether the anti-RA effect exerted by SPRC is related to H_2_S production, since SPRC can increase CSE expression, we applied HPLC-MS to detect the derivative product, sulfide dibimane (SDB), of S^2−^/HS^−^/H_2_S in rat plasma. In the standard group, Na_2_S (0–20 μM) generated SDB in a linear manner (Figure 3A,C). The retention times of SDB and internal standard (IS) hydrocortisone were about 3.0 and 3.4 min, respectively (Figure 3A,B,D,E). We found that there was a fluctuation in the model group compared to the sham group, without a significance change (Figure 3F). As shown in Figure 3F, SPRC increased total H_2_S concentration in rat plasma. These data indicate that SPRC depresses foot swelling and inhibits bone erosion in the AIA animal model, which is positively correlated with H_2_S production.

### 3.3. SPRC Decreases HDAC6 Expression and Inhibits NF-κB Signaling Pathway via Increasing CSE Expression in Synovium

**Figure 4:**
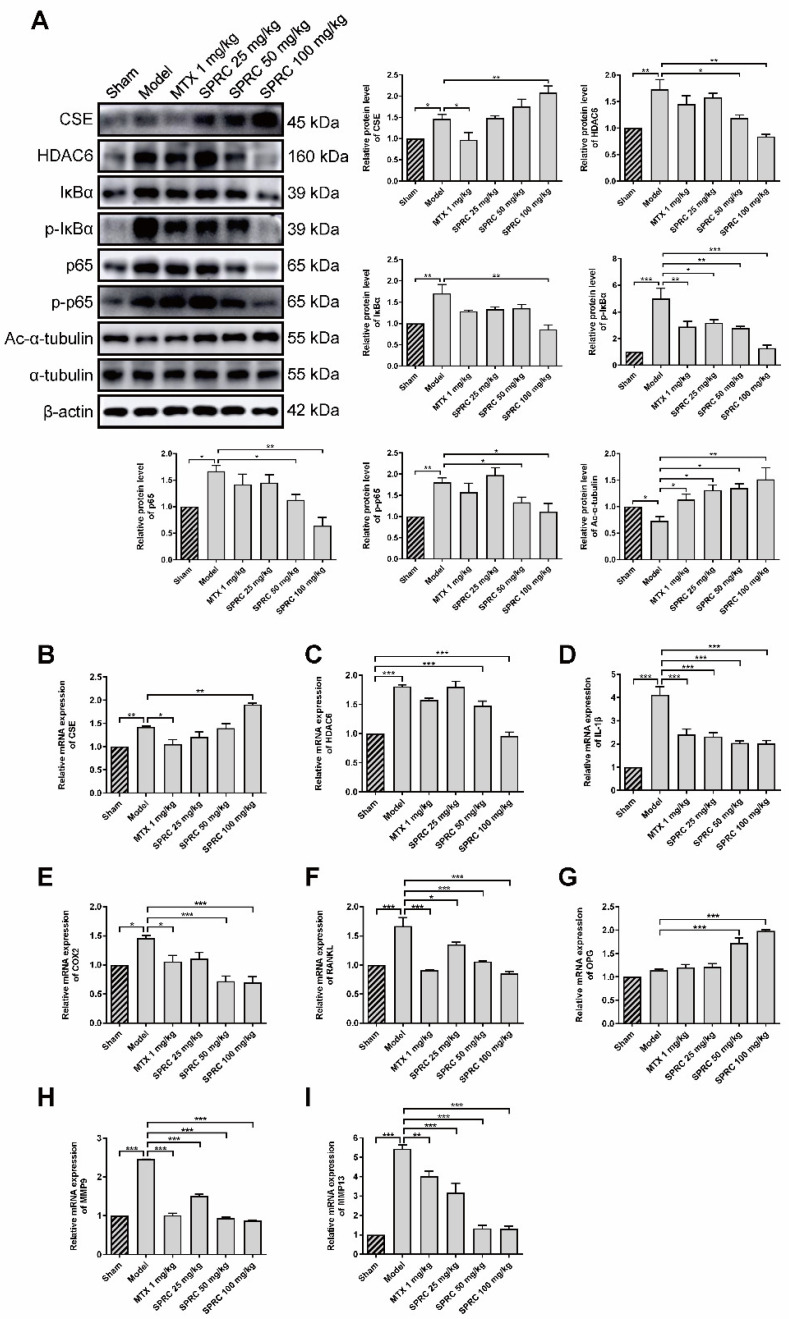
SPRC inhibits the expression of HDAC6 and inactivates NF-κB pathway in AIA rat model. (**A**) The protein levels of CSE, HDAC6, IκBα, p-IκBα, NF-κB (p65), p-NF-κB (p65), and acetylated (Ac-)α-tubulin in synovium tissues were detected by Western blotting. The gene levels of (**B**) CSE, (**C**) HDAC6, (**D**) IL-1β, (**E**) COX2, (**F**) RANKL, (**G**) OPG, (**H**) MMP9, and (**I**) MMP13 in synovium tissues were analyzed by qRT-PCR. MTX 1 mg/kg: twice a week for 30 days, intraperitoneal. SPRC 25/50/100 mg/kg: daily for 30 days, intragastric. Bars indicate the mean ± SEM. * *p* < 0.05, ** *p* < 0.01, *** *p* < 0.001. *n* = 3–4 in each group.

Since SPRC can ameliorate RA in the AIA rat model, we investigated whether SPRC had an effect on the NF-κB signaling pathway. Proteins were isolated from rat knee joint synovium and analyzed by Western blotting. SPRC significantly increased CSE expression in a dose-dependent manner (Figure 4A). However, a slight elevation in CSE level was also observed in the model group, which might be due to compensatory regulation. According to the results of previous studies, that CSE silencing increased HDAC6 mRNA level, we assumed that CSE promotion might suppress HDAC6 expression. Subsequent investigation revealed that HDAC6 was markedly increased in the model group, and down-regulated after SPRC treatment (Figure 4A), indicating that HDAC6 is a pro-RA factor and can be depressed by CSE. Meanwhile, SPRC decreased total and phosphorylated IκBα/p65 levels (Figure 4A), which indicates the inhibition of the NF-κB signaling pathway. Moreover, HDAC6 has a representative function; it can deacetylate α-tubulin [12]. Reduced levels of acetylated α-tubulin were observed in the model group as a result of HDAC6 overexpression (Figure 4A). Thereby, SPRC increased acetylated α-tubulin via suppressing HDAC6 expression.

As NF-κB can translocate into the nucleus to regulate the expression of IL-1β, COX2, and matrix metalloproteinases (MMPs) [49], we further examined whether SPRC-induced NF-κB inactivation can modulate downstream genes in the synovium, and qPCR assays were performed to analyze the expression of NF-κB targeted genes. The results show that the CSE gene was up-regulated, and the HDAC6 gene was down-regulated, after SPRC treatment (Figure 4B,C), which is consistent with previous Western blot data (Figure 4A). In addition, SPRC decreased the expression of pro-inflammatory IL-1β and COX2 (Figure 4D,E). Meanwhile, destructive bone gene RANKL was suppressed, and the bone protective gene OPG was elevated in the presence of SPRC (Figure 4F,G). MMP9 and MMP13 were previously reported to promote RA progress [50,51], and our results show that SPRC also inhibited MMP9/13 expression (Figure 4H,I). Collectively, these data demonstrate that HDAC6 promotes RA, and SPRC inhibits HDAC6- and NF-κB-related pro-RA genes, exhibiting an anti-RA effect by enhancing CSE expression in vivo.

### 3.4. H_2_S Inhibits MyD88 Deacetylation to Inactivate NF-κB Signaling Pathway Induced by HDAC6 Overexpression in rRAFLS

**Figure 5:**
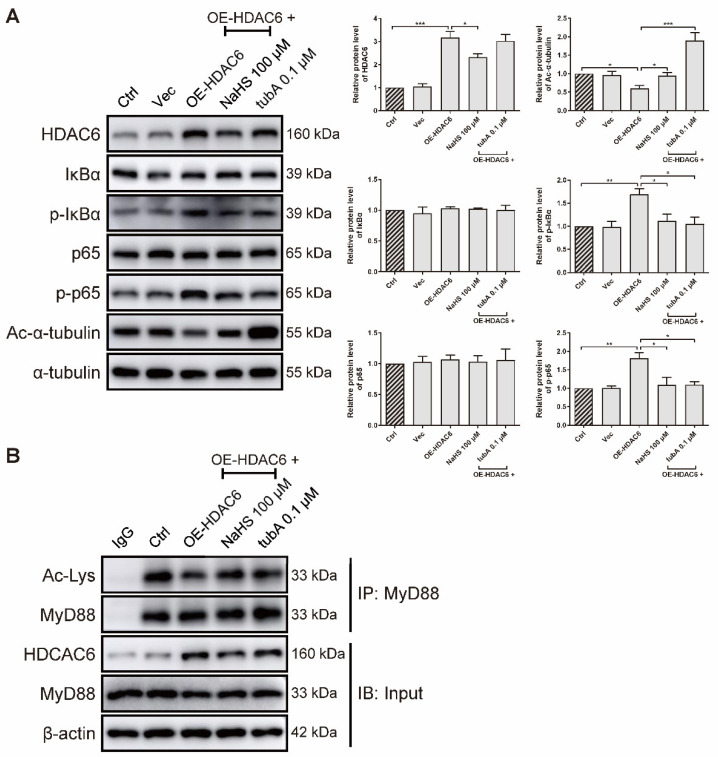
H_2_S blocks HDAC6-induced MyD88 deacetylation and NF-κB signaling activation in rRAFLS. (**A**) Immunoblot of the expression of HDAC6, Ac-α-tubulin, IκBα, p-IκBα, NF-κB (p65), and p-NF-κB (p65) in rRAFLS, which were transfected with vector (Vec) or HDAC6 overexpression plasmid OE-HDAC6 for 6 h and treated with NaHS (100 μM) or tubA (0.1 μM) for another 24 h. (**B**) After the above treatment, cell lysates were immunoprecipitated using anti-MyD88 antibody and the levels of MyD88, HDAC6, and acetylated (Ac) lysines in MyD88 were detected by Western blotting. Bars indicate the mean ± SEM. * *p* < 0.05, ** *p* < 0.01, *** *p* < 0.001. In (**A**), *n* = 3 in each group.

As forced HDAC6 expression elevated the IL-6 gene level (Figure 1I), we further examined whether HDAC6 overexpression activates the NF-κB signaling pathway at the protein level in rRAFLS. We transfected rRAFLS with an HDAC6 overexpression (OE-HDAC6) plasmid, and found that HDAC6 protein level was markedly up-regulated (Figure 5A). TubA exhibited no effect on HDAC6 protein level because tubA merely combines with the HDAC6 protein to suppress its function. NaHS reduced HDAC6 expression, while tubA did not. Consequently, NaHS and tubA elevated acetylated α-tubulin levels (Figure 5A). Although neither NaHS or tubA altered the total IκBα and p65 protein levels (Figure 5A), they inhibited the phosphorylation of IκBα and p65 (Figure 5A), which indicates that NF-κB activation induced by forced HDAC6 expression can be blocked by NaHS and tubA. Acetyl groups on MyD88 lysine residues restrict the activation of NF-κB. To confirm whether augmented NF-κB activation induced by HDAC6 overexpression is a result of MyD88 deacetylation, an IP assay was utilized to examine acetylated lysine level of MyD88. As demonstrated in Figure 5B, forced HDAC6 expression decreased acetylated MyD88 level, and NaHS/tubA reversed the modification. These data indicate that H_2_S inhibits NF-κB activation by suppressing HDAC6 expression and HDAC6-induced MyD88 deacetylation.

### 3.5. H_2_S Inhibits HDAC6 Expression by S-Sulfhydrating Transcription Factor Sp1 in rRAFLS

**Figure 6:**
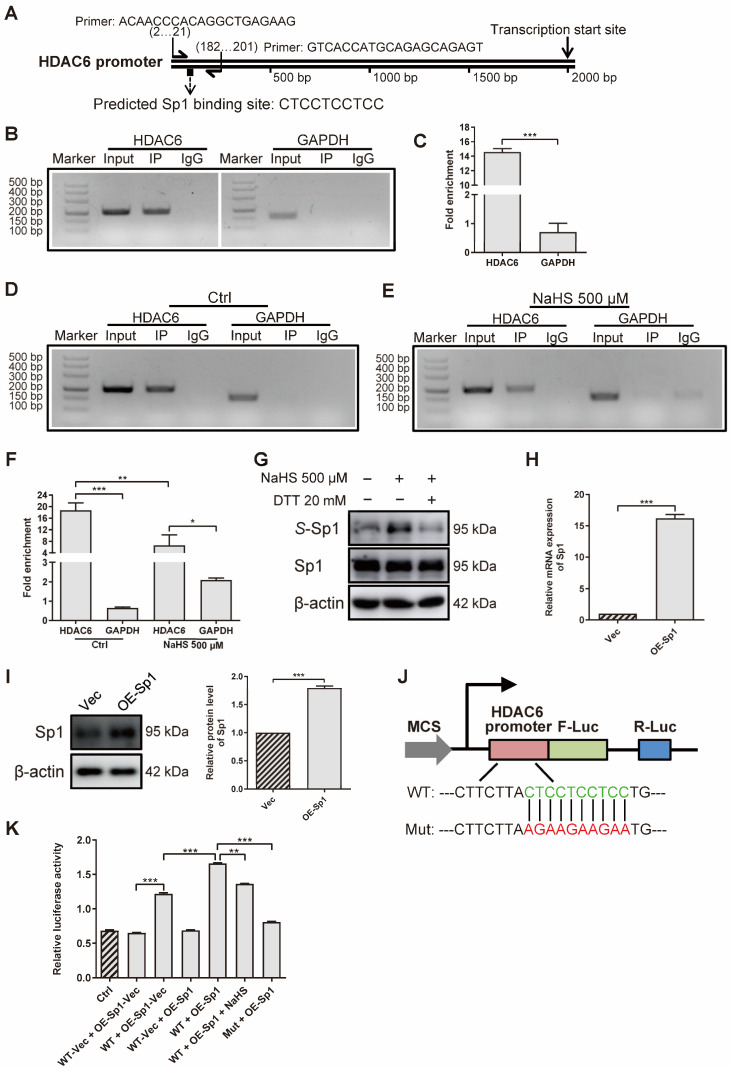
H_2_S S-sulfhydrates transcription factor Sp1 to inhibit HDAC6 expression in rRAFLS. (**A**) Predicted Sp1 binding site on HDAC6 promoter and specific HDAC6 primer. (**B**,**D**,**E**) The ChIP–PCR products were analyzed by 3% agarose gel electrophoresis. (**C**,**F**) RT-qPCR was applied to quantify fold of enrichment after IP. Cells were treated with NaHS (500 μM) for 12 h in E and F. (**G**) The protein levels of total Sp1 and S-sulfhydrated Sp1 (S-Sp1) after NaHS (30 min) or DTT (10 min) treatment were detected by Western blotting. (**H**) The gene and (**I**) protein levels of Sp1 after transfection with overexpression plasmid (OE-Sp1) for 12 h and 24 h were detected by Western blotting and RT-qPCR, respectively. (**J**) Illustration for HDAC6 wildtype (WT) and mutant (Mut) promoter cloned and inserted into plasmids. (**K**) The ratios of dual-luciferase activity were calculated after transfection with WT vector (WT-Vec), WT, or Mut of HDAC6 promoter plasmids together with Sp1 overexpression plasmid (OE-Sp1) or its negative control OE-Sp1-Vec for 6 h. Then, in WT + OE-Sp1 + NaHS group, rRAFLS were treated with NaHS (100 μM) for another 12 h. Bars indicate the mean ± SEM. * *p* < 0.05, ** *p* < 0.01, *** *p* < 0.001. In (**C**,**F**,**H**,**I**,**K**), *n* = 3 in each group.

To investigate how H_2_S decreases HDAC6 expression at gene and protein levels, we applied ChIP assays to confirm the effect of H_2_S on the HDAC6 transcription factor. First of all, we predicted several transcription factors that might bind to the HDAC6 promoter, derived from the JASPAR website (http://jaspar.genereg.net/ (accessed on 4 August 2020)) (Table S1). According to the result that LPS increased HDAC6 gene level (Figure 1F) and HDAC6 increased NF-κB phosphorylation (Figure 5A), we hypothesized that NF-κB would enhance HDAC6, as LPS mainly stimulates NF-κB and, in turn, HDAC6 augments NF-κB activation, which is a positive feedback loop. Therefore, Sp1 was chosen for further study because it not only obtains a high score, but can also combine with NF-κB to regulate gene expression [52,53]. Then, we designed a pair of HDAC6 primers to amplify specific DNA fragments pulled down by the anti-Sp1 antibody (Figure 6A). The results show that Sp1 bound to the HDAC6 promoter at the predicted site in rRAFLS (Figure 6B). The fold of enrichment being larger than four in the HDAC6 group also suggests that Sp1 could bind to the HDAC6 promoter (Figure 6C). Meanwhile, NaHS treatment decreased the combination between Sp1 and HDAC6 promoter (Figure 6D–F). Subsequently, we examined whether reduced Sp1 combination with HDAC6 promoter is induced by S-sulfhydration. As shown in Figure 6G, NaHS increased the S-sulfhydrated Sp1 level, and the reductant DTT reversed the change. Meanwhile, overexpressed Sp1 (Figure 6H,I) can combine with the HDAC6 promoter at the indicated site (Figure 6J,K). NaHS (100 μM) treatment, and the mutation of the binding site, inhibited the initiation of HDAC6 transcription, which further confirmed that Sp1 can combine with the HDAC6 promoter at -CTCCTCCTCC- site. Collectively, H_2_S S-sulfhydrates HDAC6 transcription factor Sp1 to restrict their combination, and HDAC6 expression, in rRAFLS.

## 4. Discussion

In RA, a number of immune cells, such as T cells, B cells, and macrophages, infiltrate the synovial sublining and stimulate other effector cells, including synoviocytes, chondrocytes, as well as osteoclasts [54]. Subsequently, cytokines, chemokines, growth factors, cellular ligands, adhesion molecules, and transcription factors produced by these cells trigger the deleterious process of joint destruction. Although RA is a systemic disease, the synovium plays a central role in RA progress since synovial lining fibroblasts contribute to the local inflammatory feedback loops, which are composed of excessive cytokines and proteolytic enzymes [4]. It has been reported that the dominant destructive cell type for cartilage is considered to be the cadherin-11^+^ FLS [55]. In our study, we applied primary FLS as the cell model to perform different assays, which, although reasonable, was not sufficient. H_2_S is a lipophilic molecule that can penetrate cell membranes without the requirement of any specific transporter [56], suggesting that it may simultaneously affect other cell types in RA treatment. Although H_2_S can inhibit immune-related inflammatory responses in rRAFLS, we need to explore more anti-RA mechanisms of H_2_S in immune cells, such as dendritic cells, T cells, and/or B cells.

Our previous results indicated that CSE/H_2_S inhibits JMJD3 to induce an anti-RA effect in CIA mice [57]; this was the first study to reveal the association between H_2_S, RA, and epigenetics. In the present study, the anti-RA effect of exogenous H_2_S donor SPRC was again confirmed, which is consistent with the work of another group [41]. In their report, SPRC showed antioxidative efficacy in the AIA rat model by regulating the Nrf2/HO-1 signaling pathway, resulting in blocked RA progress. However, we revealed another novel mechanism, that SPRC/H_2_S exerted anti-RA activity by inhibiting a controversial epigenetic enzyme HDAC6 in a AIA rat model. Therefore, H_2_S can regulate not only histone demethylase, but also histone deacetylase. It was reported that the inhibition of HDAC6 exerted an anti-RA effect in vitro and in vivo [22,58]. Moreover, the inhibition of HDAC6 attenuated LPS-induced inflammation in macrophages and the nervous system [20,59]. In our study, the data support the concept that HDAC6 promotes inflammatory responses in RAFLS, as HDAC6 was highly expressed in the AIA model and overexpression of HDAC6 activated the NF-κB signaling pathway. Consequently, HDAC6 inhibition might be an effective strategy in RA treatment due to its role in the regulation of inflammation. For example, in the CIA mouse model and RA patients, the HDAC6 selective inhibitor, CKD-L, inhibits IL-6, TNF-α, and IL-1β expression, and increases IL-10 production, resulting in a decreased arthritis score and inhibited proliferation of effector T cells [22]. In addition, the novel HDAC6 inhibitor, CKD-506, suppresses inflammatory responses by monocytes/macrophages, improves the function of regulatory T cells, and ameliorates arthritis severity in AIA rats [58]. A recently developed HDAC6 inhibitor, M-134, increases the efficacy of tofacitinib by regulating various cytokines, such as IL-1β, IL-17, and TNF-α, in the serum of AIA rats [60]. To our surprise, the expression of HDAC6 in RA patients remained unchanged compared with OA patients [61]. Therefore, in future experiments we will collect clinical samples to analyze the change of HDAC6 expression. The fact that CSE silencing elevated HDAC6 expression, and SPRC/H_2_S treatment decreased HDAC6 expression, at both gene and protein levels, confirms that H_2_S can inhibit HDAC6 gene expression and, consequently, reduce HDAC6 protein level. As a transcription factor, Sp1 was proven, for the first time, to regulate HDAC6 gene expression. Therefore, targeting Sp1 might be a potential therapeutic strategy for RA treatment.

S-sulfhydration on proteins was first described in 2009 [62]; it is a post-translational modification (PTM) initiated by forming a hydropersulfide moiety (-SSH) in cysteine residues. Several reports support the idea that H_2_S can regulate proteins through S-sulfhydration [44,62]. This modification is related to the physiological and pathological functions of H_2_S [63,64]. S-sulfhydration of Kelch-like ECH-associated protein 1 (Keap1) provides protection against cellular senescence by regulating Nrf2 activity [44]. It was also found that S-sulfhydration of mitogen-activated protein kinase 1 (MEK1) is associated with repairing damaged DNA inside the cell [65]. In addition, S-sulfhydration of ATP synthase by H_2_S stimulates mitochondrial bioenergetics [66]. Although H_2_S has been reported to have an anti-inflammation effect [67,68], the exact underlying mechanisms regarding how H_2_S acts on target proteins have not been elucidated. In our study, we directly detected the S-sulfhydration level of Sp1 in the presence of H_2_S, and found that H_2_S promoted Sp1 S-sulfhydration. Hence, S-sulfhydrated Sp1 could not bind to the HDAC6 promoter efficiently and regulate its transcription. This provides evidence that H_2_S inhibited HDAC6 expression by S-sulfhydrating its transcription factor Sp1.

Since p53 was reported as a non-histone protein that can be acetylated by HAT [69], there has been a rapid proliferation in the study of other non-histone targets of histone acetyltransferases (HATs) and HDACs, such as STAT3, c-MYC, NF-κB, α-tubulin, and so on [70]. The acetylation and deacetylation of non-histone proteins plays various roles in diverse human diseases, including cancer, RA, and Parkinson’s disease (PD) [71,72,73]. MyD88 is a non-histone protein that can be directly acetylated or deacetylated, and this modification impacts its biological properties [14]. MyD88 is an essential cytosolic adaptor protein for signaling induced by members of the TLR and IL-1R family, which play an important role in the production of diverse pro-inflammatory cytokines through the activation of NF-κB [74,75]. The deletion of MyD88 was found to result in a significant reduction in the secretion of inflammatory cytokines [76]. In human primary pulmonary microvascular endothelial cells, HDAC6 inhibition augmented the acetylation of MyD88 induced by LPS, promoted the combination of MyD88 and TRAF6, and enhanced inflammation [13]. In U2OS cells, the deacetylation of lysine residues of MyD88 generated by HDAC6 directly inhibited MyD88 activity, resulting in decreased cytokine levels controlled by MyD88-dependent TLR signaling [14]. As an anti-rheumatic agent, sinomenine inhibited the TLR4/MyD88/NF-κB signaling pathway to prevent IL-1β-induced inflammation in human RAFLS [77]. Similarly, baicalin treatment markedly decreased the gene expression of MyD88 and TLR2, as well as the protein levels of MyD88, TLR2, and NF-κB p65 in human RAFLS [78]. Therefore, the activation of the NF-κB signaling pathway induced by MyD88 contributes to RA progression. In this study, we confirm that HDAC6 can deacetylate MyD88 to inhibit the NF-κB signaling pathway in RAFLS. Therefore, we clarify how HDAC6 affects the NF-κB signaling pathway.

## 5. Conclusions and Limitations

In conclusion, H_2_S S-sulfhydrates Sp1 to down-regulate HDAC6 expression, then the deacetylation of MyD88 regulated by HDAC6 is decreased, and downstream NF-κB activation is restrained. Thereby, H_2_S ameliorates the inflammatory response in AIA rats, which provides further evidence of the anti-RA properties of H_2_S, and the potential for H_2_S donor (such as SPRC) development in RA therapy. However, the S-sulfhydration sites of Sp1 were not detected in this study. This information is essential, and we will confirm the exact amino acid structure modified by H_2_S in further study.

## Data Availability

All data needed to evaluate the conclusions in the paper are present in the paper and the Supplementary Materials.

## Supplementary Materials

The following supporting information can be downloaded at: https://www.mdpi.com/article/10.3390/antiox11040732/s1, Figure S1: CSE silencing decreases HDAC6 expression in rRAFLS; Table S1: Predicted transcription factors that might bind to HDAC6 promoter.
