# Supplementary material for: Sp1 S-Sulfhydration Induced by Hydrogen Sulfide Inhibits Inflammation via HDAC6/MyD88/NF-κB Signaling Pathway in Adjuvant-Induced Arthritis
Source: Antioxidants (Basel). 2022 Apr 7;11(4):732. doi: 10.3390/antiox11040732 (PMC9030249; doi:10.3390/antiox11040732)
Supplement: Supplementary file 1 [file antioxidants-11-00732-s001.zip › Supplementary Figure S1.pdf]

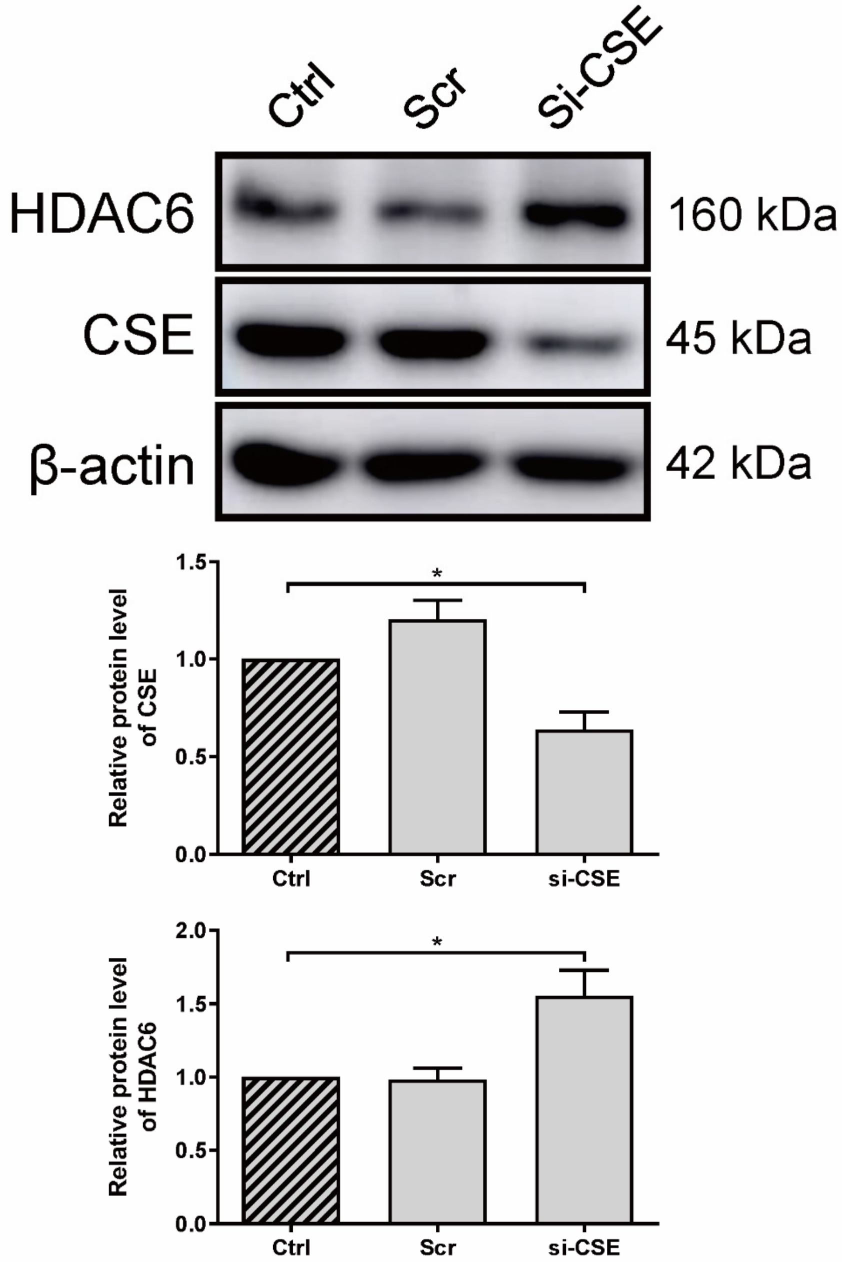

Figure S1. CSE silencing decreases HDAC6 expression in rRAFLS. The protein levels of CSE and HDAC6 were detected by western blotting. *$p < 0.05$. n = 3 in each group.
