# Supplementary material for: Sp1 S-Sulfhydration Induced by Hydrogen Sulfide Inhibits Inflammation via HDAC6/MyD88/NF-κB Signaling Pathway in Adjuvant-Induced Arthritis
Source: Antioxidants (Basel). 2022 Apr 7;11(4):732. doi: 10.3390/antiox11040732 (PMC9030249; doi:10.3390/antiox11040732)
Supplement: Supplementary file 1 [file antioxidants-11-00732-s001.zip › Supplementary Table S1.pdf]

Table S1. Predicted transcription factors of HDAC6

Matrix ID	Name	Score	Relative	Sequence	Start	End	Strand	Predicted sequence
MA0041.1	Foxd3	12.4852	0.918654	HDAC6	374	385	+	ATTTATTTATTT
MA0156.1	FEV	12.0467	1	HDAC6	1411	1418	+	CAGGAAAT
MA0041.1	Foxd3	11.9999	0.908971	HDAC6	547	558	+	GATTTTTTTTTT
MA0047.1	Foxa2	11.7592	0.925347	HDAC6	374	385	+	ATTTATTTATTT
MA0152.1	NFATC2	11.3597	1	HDAC6	1173	1179	+	TTTTCCA
MA0041.1	Foxd3	10.6328	0.881697	HDAC6	740	751	+	TATTATTTTCTT
MA0079.2	SP1	10.5047	0.895848	HDAC6	105	114	+	CTCCTCCTCC
MA0007.1	Ar	10.3835	0.805297	HDAC6	684	705	+	CATGGGACTTCATGTTCTACAG
MA0047.1	Foxa2	10.2843	0.887573	HDAC6	370	381	+	AAAGATTTATTT
MA0156.1	FEV	10.0806	0.931247	HDAC6	569	576	+	AAGGAAGT
MA0160.1	NR4A2	10.0359	0.943686	HDAC6	705	712	-	AAGATCAC
MA0041.1	Foxd3	9.95163	0.868108	HDAC6	548	559	+	ATTTTTTTTTTT
MA0079.2	SP1	9.7793	0.87758	HDAC6	1317	1326	+	CCCCACCCCT
MA0040.1	Foxq1	9.7765	0.852345	HDAC6	633	643	+	TAAAGTTTACT
MA0156.1	FEV	9.66612	0.916754	HDAC6	936	943	+	TAGGAAAT
MA0079.2	SP1	9.53812	0.871506	HDAC6	1697	1706	-	CCCCACTCCC
MA0079.2	SP1	9.50209	0.870599	HDAC6	1684	1693	-	CCCCGCATCC
MA0160.1	NR4A2	9.42687	0.922285	HDAC6	1204	1211	-	CAGGTCAC
MA0041.1	Foxd3	9.40661	0.857235	HDAC6	549	560	+	TTTTTTTTTTTT
MA0038.1	Gfi1	9.32388	0.902148	HDAC6	1220	1229	+	TAAATCTAAG
MA0038.1	Gfi1	9.30717	0.901646	HDAC6	712	721	+	TCAATCTCTC
MA0113.1	NR3C1	9.18755	0.813234	HDAC6	1124	1141	+	AGAAACAGTCCTTTGCAA
MA0152.1	NFATC2	9.18689	0.919862	HDAC6	1047	1053	+	TTTTCCC
MA0040.1	Foxq1	9.18075	0.837039	HDAC6	1419	1429	-	AAATGTTGATA
MA0116.1	Znf423	9.09193	0.842274	HDAC6	139	153	-	AGAGCCTAAGGAGGC
MA0038.1	Gfi1	9.072	0.894581	HDAC6	617	626	+	GAAATCTGAG
MA0113.1	NR3C1	9.03803	0.810305	HDAC6	1120	1137	+	CAGGAGAAACAGTCCTTT
MA0079.2	SP1	8.93679	0.856362	HDAC6	1696	1705	-	CCCACTCCCC
MA0041.1	Foxd3	8.85338	0.846198	HDAC6	370	381	+	AAAGATTTATTT
MA0047.1	Foxa2	8.83578	0.850475	HDAC6	342	353	-	AACCATTTGTTC
MA0047.1	Foxa2	8.82536	0.850208	HDAC6	740	751	+	TATTATTTTCTT
MA0113.1	NR3C1	8.8111	0.80586	HDAC6	503	520	+	AAGAGCAGACTGCTCTTA
MA0041.1	Foxd3	8.79193	0.844972	HDAC6	326	337	-	AATTATTTTTTG
MA0099.2	FOS::JUN	8.77298	0.930975	HDAC6	1296	1302	+	TGACACA
MA0099.2	FOS::JUN	8.68023	0.92759	HDAC6	1084	1090	+	TTACTCA
MA0442.1	SOX10	8.62523	0.987367	HDAC6	1262	1267	-	CATTGT
MA0152.1	NFATC2	8.59374	0.897985	HDAC6	1556	1562	+	TCTTCCA
MA0152.1	NFATC2	8.59374	0.897985	HDAC6	340	346	-	TGTTCCA
MA0152.1	NFATC2	8.59374	0.897985	HDAC6	500	506	-	TCTTCCA
MA0259.1	ARNT::HIF	8.57453	0.921705	HDAC6	200	207	+	ACACGTGT
MA0259.1	ARNT::HIF	8.57453	0.921705	HDAC6	200	207	-	ACACGTGT
MA0018.2	CREB1	8.56662	0.890064	HDAC6	254	261	+	TGAGGCCA
MA0160.1	NR4A2	8.55354	0.891599	HDAC6	945	952	-	GAGGACAC
MA0160.1	NR4A2	8.4272	0.887161	HDAC6	962	969	+	AAGATCAG
MA0160.1	NR4A2	8.4272	0.887161	HDAC6	1149	1156	-	AAGATCAG
MA0259.1	ARNT::HIF	8.40904	0.916768	HDAC6	798	805	+	GAACGTGG
MA0079.2	SP1	8.31578	0.840722	HDAC6	1860	1869	-	ACCCGCCCAC
MA0079.2	SP1	8.29848	0.840287	HDAC6	1689	1698	-	CCCACCCCCG
MA0099.2	FOS::JUN	8.18522	0.909521	HDAC6	1273	1279	+	TGCCTCA
MA0041.1	Foxd3	8.17701	0.832704	HDAC6	1074	1085	+	ATTAGTTTGATT
MA0152.1	NFATC2	8.13433	0.881041	HDAC6	1965	1971	-	CTTTCCA
MA0018.2	CREB1	8.12996	0.874073	HDAC6	438	445	-	TGAGGTCT
MA0047.1	Foxa2	8.09904	0.831606	HDAC6	378	389	+	ATTTATTTATTA
MA0041.1	Foxd3	8.09608	0.831089	HDAC6	32	43	+	AATTGTTGGACT
MA0041.1	Foxd3	8.06426	0.830455	HDAC6	378	389	+	ATTTATTTATTA
MA0041.1	Foxd3	8.04494	0.830069	HDAC6	901	912	+	GATGGTTTCTTT
MA0040.1	Foxq1	7.91388	0.80449	HDAC6	374	384	+	ATTTATTTATT
MA0040.1	Foxq1	7.91388	0.80449	HDAC6	378	388	+	ATTTATTTATT
MA0156.1	FEV	7.90454	0.855153	HDAC6	1983	1990	+	CAGTAAGT

MA0156.1	FEV	7.90454	0.855153	HDAC6	690	697	-	CATGAAGT
MA0040.1	Foxq1	7.90116	0.804163	HDAC6	1257	1267	-	CATTGTTAAGG
MA0079.2	SP1	7.85067	0.829009	HDAC6	1971	1980	-	CACTGCCCCC
MA0019.1	Ddit3::Ce	7.82989	0.816225	HDAC6	687	698	-	ACATGAAGTCCC
MA0018.2	CREB1	7.82898	0.863051	HDAC6	1510	1517	-	TGACGTAG
MA0041.1	Foxd3	7.79486	0.82508	HDAC6	357	368	+	GCTTGATTTTTT
MA0079.2	SP1	7.76117	0.826755	HDAC6	225	234	-	CTCTGTCCCC
MA0060.1	NFYA	7.72264	0.809498	HDAC6	1575	1590	-	TGGAGCAAATCAAAGA
MA0019.1	Ddit3::Ce	7.71646	0.813397	HDAC6	846	857	-	TAATCCAATAGC
MA0160.1	NR4A2	7.6295	0.859132	HDAC6	659	666	-	AAGGTCCC
MA0105.1	NFKB1	7.62925	0.820317	HDAC6	687	696	+	GGGACTTCAT
MA0099.2	FOS::JUN	7.61448	0.888688	HDAC6	670	676	+	TGACTCT
MA0099.2	FOS::JUN	7.61448	0.888688	HDAC6	1031	1037	-	TGACTCT
MA0099.2	FOS::JUN	7.61448	0.888688	HDAC6	1947	1953	-	TGACTCG
MA0259.1	ARNT::HIF	7.41631	0.887152	HDAC6	1758	1765	-	CTGCGTGG
MA0102.2	CEBPA	7.38484	0.861773	HDAC6	1642	1650	-	ATTCTCAAT
MA0117.1	Mafb	7.3721	0.907874	HDAC6	1652	1659	-	GCTGCCGC
MA0038.1	Gfi1	7.37022	0.843459	HDAC6	369	378	-	TAAATCTTTA
MA0160.1	NR4A2	7.36879	0.849972	HDAC6	136	143	-	GAGGCCAA
MA0117.1	Mafb	7.31731	0.905502	HDAC6	809	816	+	GCTGAAAC
MA0102.1	Cebpa	7.28869	0.835128	HDAC6	613	624	+	ATTTGAAATCTG
MA0079.2	SP1	7.22792	0.813325	HDAC6	1953	1962	-	CTCCGCCCTT
MA0079.2	SP1	7.21446	0.812986	HDAC6	1856	1865	-	GCCCACCTCC
MA0079.2	SP1	7.21216	0.812928	HDAC6	1695	1704	-	CCACTCCCCA
MA0152.1	NFATC2	7.17492	0.845656	HDAC6	937	943	-	ATTTCCT
MA0152.1	NFATC2	7.17492	0.845656	HDAC6	1412	1418	-	ATTTCCT
MA0079.2	SP1	7.17191	0.811915	HDAC6	1849	1858	-	TCCAGCCCAC
MA0105.1	NFKB1	7.15825	0.808911	HDAC6	300	309	-	GGGTCTCTCC
MA0041.1	Foxd3	7.10875	0.811392	HDAC6	544	555	+	CATGATTTTTTT
MA0442.1	SOX10	7.09699	0.919521	HDAC6	1235	1240	+	CTGTGT
MA0442.1	SOX10	7.09699	0.919521	HDAC6	163	168	-	CTGTGT
MA0442.1	SOX10	7.09699	0.919521	HDAC6	1011	1016	-	CTGTGT
MA0442.1	SOX10	7.09699	0.919521	HDAC6	1920	1925	-	CTGTGT
MA0442.1	SOX10	7.09357	0.919369	HDAC6	1823	1828	+	CGTTGT
MA0099.2	FOS::JUN	7.07754	0.869089	HDAC6	886	892	-	TGCATCA
MA0038.1	Gfi1	7.05956	0.834126	HDAC6	543	552	-	AAAATCATGG
MA0079.2	SP1	7.0594	0.809081	HDAC6	1692	1701	-	CTCCCCACCC
MA0079.2	SP1	7.02995	0.80834	HDAC6	1966	1975	-	CCCCCTTTCC
MA0079.2	SP1	6.99949	0.807572	HDAC6	1965	1974	-	CCCCTTTCCA
MA0102.1	Cebpa	6.99219	0.825951	HDAC6	1639	1650	-	ATTCTCAATTCC
MA0041.1	Foxd3	6.99194	0.809061	HDAC6	329	340	-	AAAAATTATTTT
MA0038.1	Gfi1	6.95455	0.830972	HDAC6	1139	1148	+	CAAATCCATT
MA0160.1	NR4A2	6.94479	0.835074	HDAC6	255	262	+	GAGGCCAG
MA0160.1	NR4A2	6.94479	0.835074	HDAC6	1192	1199	+	GAGGACAG
MA0152.1	NFATC2	6.94041	0.837007	HDAC6	1427	1433	+	TTTACCA
MA0116.1	Znf423	6.90905	0.805793	HDAC6	217	231	+	AGAACTCAGGGGACA
MA0160.1	NR4A2	6.89478	0.833317	HDAC6	771	778	-	AAGGGCAA
MA0038.1	Gfi1	6.86688	0.828338	HDAC6	759	768	-	TAAATCTACA
MA0116.1	Znf423	6.85172	0.804835	HDAC6	1535	1549	+	GGAATCTTGGGAGCA
MA0442.1	SOX10	6.81243	0.906887	HDAC6	399	404	-	CAGTGT
MA0442.1	SOX10	6.81243	0.906887	HDAC6	1300	1305	-	CAGTGT
MA0079.2	SP1	6.77514	0.801922	HDAC6	1322	1331	+	CCCCTGCACC
MA0160.1	NR4A2	6.76845	0.828878	HDAC6	484	491	-	AAGTTCAA
MA0038.1	Gfi1	6.76053	0.825143	HDAC6	22	31	-	TAAAACAGTG
MA0041.1	Foxd3	6.7597	0.804428	HDAC6	1572	1583	+	GCTTCTTTGATT
MA0156.1	FEV	6.75612	0.814994	HDAC6	1930	1937	+	CCGGGAAT
MA0156.1	FEV	6.7085	0.813329	HDAC6	1564	1571	-	TAGGATGT
MA0041.1	Foxd3	6.70552	0.803347	HDAC6	1078	1089	+	GTTTGATTACTC
MA0099.2	FOS::JUN	6.69649	0.85518	HDAC6	1982	1988	-	TTACTGA
MA0038.1	Gfi1	6.65657	0.82202	HDAC6	703	712	-	AAGATCACTG

MA0099.2	FOS::JUN	6.65634	0.853715	HDAC6	50	56	+	TGGCTCA
MA0099.2	FOS::JUN	6.65634	0.853715	HDAC6	310	316	+	TGTCTCA
MA0099.2	FOS::JUN	6.65634	0.853715	HDAC6	459	465	-	TGGCTCA
MA0099.2	FOS::JUN	6.65634	0.853715	HDAC6	526	532	-	TGGCTCA
MA0099.2	FOS::JUN	6.65634	0.853715	HDAC6	1304	1310	-	TGGCTCA
MA0041.1	Foxd3	6.65431	0.802326	HDAC6	1360	1371	-	AAATGTTAGCTA
MA0442.1	SOX10	6.63642	0.899073	HDAC6	131	136	+	CTTTCT
MA0442.1	SOX10	6.63642	0.899073	HDAC6	909	914	+	CTTTCT
MA0442.1	SOX10	6.63642	0.899073	HDAC6	1291	1296	+	CTTTCT
MA0442.1	SOX10	6.63642	0.899073	HDAC6	1845	1850	+	TTTTGT
MA0442.1	SOX10	6.63642	0.899073	HDAC6	562	567	-	CTTTCT
MA0442.1	SOX10	6.63642	0.899073	HDAC6	566	571	-	CTTTCT
MA0442.1	SOX10	6.63642	0.899073	HDAC6	782	787	-	TTTTGT
MA0442.1	SOX10	6.63642	0.899073	HDAC6	925	930	-	CTTTCT
MA0442.1	SOX10	6.63642	0.899073	HDAC6	1995	2000	-	TTTTGT
MA0038.1	Gfi1	6.58107	0.819752	HDAC6	1804	1813	-	CAAACCACAC
MA0041.1	Foxd3	6.54939	0.800232	HDAC6	737	748	+	TAGTATTATTTT
MA0156.1	FEV	6.53424	0.807235	HDAC6	1931	1938	+	CGGGAATT
MA0018.2	CREB1	6.52875	0.815434	HDAC6	1650	1657	-	TGCCGCCA
MA0038.1	Gfi1	6.4772	0.816631	HDAC6	1437	1446	-	CAAATGAGTG
MA0160.1	NR4A2	6.47078	0.818419	HDAC6	126	133	-	AAGGACAT
MA0102.1	Cebpa	6.45733	0.809397	HDAC6	935	946	+	TTAGGAAATGGT
MA0160.1	NR4A2	6.44476	0.817504	HDAC6	492	499	-	GAGGTCCC
MA0102.2	CEBPA	6.4421	0.827814	HDAC6	935	943	+	TTAGGAAAT
MA0018.2	CREB1	6.43722	0.812082	HDAC6	75	82	+	TCACATCA
MA0156.1	FEV	6.43658	0.80382	HDAC6	126	133	-	AAGGACAT
MA0152.1	NFATC2	6.42094	0.817847	HDAC6	959	965	-	TCTTCCC
MA0259.1	ARNT::HIF	6.39079	0.856558	HDAC6	1706	1713	+	GAGCGTGT
MA0038.1	Gfi1	6.37938	0.813693	HDAC6	357	366	-	AAAATCAAGC
MA0018.2	CREB1	6.3593	0.809229	HDAC6	1191	1198	+	TGAGGACA
MA0018.2	CREB1	6.3593	0.809229	HDAC6	1549	1556	+	AGACGTCT
MA0018.2	CREB1	6.3593	0.809229	HDAC6	946	953	-	TGAGGACA
MA0018.2	CREB1	6.3593	0.809229	HDAC6	1549	1556	-	AGACGTCT
MA0442.1	SOX10	6.35185	0.88644	HDAC6	1145	1150	+	CATTCT
MA0442.1	SOX10	6.35185	0.88644	HDAC6	46	51	-	CATTCT
MA0442.1	SOX10	6.35185	0.88644	HDAC6	1646	1651	-	CATTCT
MA0160.1	NR4A2	6.34445	0.81398	HDAC6	622	629	-	AAGCTCAG
MA0160.1	NR4A2	6.34173	0.813885	HDAC6	967	974	+	CAGGACAA
MA0102.2	CEBPA	6.3294	0.823754	HDAC6	1962	1970	-	TTTCCAAAC
MA0156.1	FEV	6.32888	0.800054	HDAC6	744	751	-	AAGAAAAT
MA0117.1	Mafb	6.30379	0.86161	HDAC6	1189	1196	+	GCTGAGGA
MA0018.2	CREB1	6.21952	0.80411	HDAC6	1281	1288	-	TGACTTCT
MA0160.1	NR4A2	6.2154	0.809446	HDAC6	1837	1844	+	TAGATCAG
MA0117.1	Mafb	6.19577	0.856932	HDAC6	1655	1662	-	GCTGCTGC
MA0102.1	Cebpa	6.19359	0.801234	HDAC6	1417	1428	+	ATTATCAACATT
MA0117.1	Mafb	6.19059	0.856708	HDAC6	1512	1519	-	GATGACGT
MA0038.1	Gfi1	6.18719	0.80792	HDAC6	765	774	-	GCAATCTAAA
MA0038.1	Gfi1	6.16302	0.807194	HDAC6	581	590	-	TTAATCTTTG
MA0018.2	CREB1	6.14073	0.801224	HDAC6	137	144	-	GGAGGCCA
MA0102.2	CEBPA	6.11556	0.816051	HDAC6	613	621	+	ATTTGAAAT
MA0099.2	FOS::JUN	6.10893	0.833733	HDAC6	310	316	-	TGAGACA
MA0038.1	Gfi1	6.06258	0.804176	HDAC6	622	631	-	AAAAGCTCAG
MA0152.1	NFATC2	6.01624	0.802921	HDAC6	1121	1127	-	TTCTCCT
MA0160.1	NR4A2	5.99777	0.801799	HDAC6	583	590	+	AAGATTAA
MA0038.1	Gfi1	5.95273	0.800876	HDAC6	1607	1616	+	CGAATCCGTA
MA0038.1	Gfi1	5.93371	0.800305	HDAC6	76	85	+	CACATCAAAG
MA0102.2	CEBPA	5.92287	0.80911	HDAC6	337	345	+	TTTTGGAAC
MA0442.1	SOX10	5.84552	0.863962	HDAC6	1233	1238	+	CTCTGT
MA0442.1	SOX10	5.84552	0.863962	HDAC6	229	234	-	CTCTGT
MA0442.1	SOX10	5.84552	0.863962	HDAC6	271	276	-	CTCTGT

MA0442.1	SOX10	5.84552	0.863962	HDAC6	1196	1201	-	CTCTGT
MA0442.1	SOX10	5.84552	0.863962	HDAC6	1248	1253	-	CTCTGT
MA0117.1	Mafb	5.77364	0.838651	HDAC6	196	203	+	GGTGACAC
MA0117.1	Mafb	5.77364	0.838651	HDAC6	1710	1717	-	GCTAACAC
MA0117.1	Mafb	5.75593	0.837884	HDAC6	1499	1506	+	GCTGGCGC
MA0099.2	FOS::JUN	5.72348	0.819664	HDAC6	198	204	+	TGACACG
MA0099.2	FOS::JUN	5.68155	0.818133	HDAC6	207	213	-	TGAAAGA
MA0117.1	Mafb	5.60697	0.831433	HDAC6	1673	1680	-	GCTGCTGT
MA0259.1	ARNT::HIF	5.59293	0.832756	HDAC6	1783	1790	-	GCCCGTGG
MA0442.1	SOX10	5.56096	0.851329	HDAC6	22	27	+	CACTGT
MA0442.1	SOX10	5.56096	0.851329	HDAC6	400	405	+	CACTGT
MA0442.1	SOX10	5.56096	0.851329	HDAC6	702	707	-	CACTGT
MA0442.1	SOX10	5.55851	0.85122	HDAC6	1459	1464	-	CTTTGG
MA0099.2	FOS::JUN	5.54866	0.813282	HDAC6	576	582	+	TGTATCA
MA0099.2	FOS::JUN	5.54866	0.813282	HDAC6	85	91	-	TGAATCC
MA0117.1	Mafb	5.51425	0.827417	HDAC6	1375	1382	+	GCTGCTTC
MA0117.1	Mafb	5.50907	0.827193	HDAC6	407	414	+	GCTGTCTT
MA0117.1	Mafb	5.29803	0.818054	HDAC6	13	20	+	GCTGAGAA
MA0117.1	Mafb	5.29803	0.818054	HDAC6	793	800	+	GCTGAGAA
MA0117.1	Mafb	5.29413	0.817885	HDAC6	524	531	+	GCTGAGCC
MA0117.1	Mafb	5.29413	0.817885	HDAC6	1879	1886	-	GCTGAGCG
MA0117.1	Mafb	5.2829	0.817399	HDAC6	1547	1554	+	GCAGACGT
MA0442.1	SOX10	5.27395	0.838587	HDAC6	1887	1892	-	CATTGG
MA0117.1	Mafb	5.20595	0.814066	HDAC6	1831	1838	+	GCGGACTA
MA0117.1	Mafb	5.19018	0.813383	HDAC6	507	514	+	GCAGACTG
MA0117.1	Mafb	5.18483	0.813152	HDAC6	1362	1369	+	GCTAACAT
MA0117.1	Mafb	5.18483	0.813152	HDAC6	176	183	-	GTTGACAT
MA0117.1	Mafb	5.09018	0.809052	HDAC6	1627	1634	+	GATGACTA
MA0117.1	Mafb	5.09018	0.809052	HDAC6	1490	1497	-	GGTGACTA
MA0259.1	ARNT::HIF	4.93551	0.813144	HDAC6	871	878	+	CTACTTGC
MA0259.1	ARNT::HIF	4.84683	0.810498	HDAC6	1065	1072	+	CCACTTGC
MA0259.1	ARNT::HIF	4.84683	0.810498	HDAC6	1783	1790	+	CCACGGGC
MA0259.1	ARNT::HIF	4.84683	0.810498	HDAC6	235	242	-	CCACCTGC
MA0259.1	ARNT::HIF	4.84683	0.810498	HDAC6	798	805	-	CCACGTTC
MA0442.1	SOX10	4.82361	0.818594	HDAC6	309	314	+	CTGTCT
MA0442.1	SOX10	4.82361	0.818594	HDAC6	408	413	+	CTGTCT
MA0442.1	SOX10	4.82361	0.818594	HDAC6	450	455	-	CTGTCT
MA0442.1	SOX10	4.82361	0.818594	HDAC6	1590	1595	-	TTGTGT
MA0442.1	SOX10	4.82361	0.818594	HDAC6	1595	1600	-	TTGTGT
MA0442.1	SOX10	4.82019	0.818442	HDAC6	650	655	+	TGTTGT
MA0442.1	SOX10	4.82019	0.818442	HDAC6	1052	1057	+	CCTTCT
MA0442.1	SOX10	4.82019	0.818442	HDAC6	1240	1245	+	TGTTGT
MA0442.1	SOX10	4.82019	0.818442	HDAC6	1346	1351	+	CCTTCT
MA0442.1	SOX10	4.82019	0.818442	HDAC6	677	682	-	TCTTGT
MA0442.1	SOX10	4.82019	0.818442	HDAC6	797	802	-	CGTTCT
MA0442.1	SOX10	4.82019	0.818442	HDAC6	1592	1597	-	TGTTGT
MA0442.1	SOX10	4.82019	0.818442	HDAC6	1897	1902	-	CCTTCT
MA0442.1	SOX10	4.80518	0.817776	HDAC6	627	632	+	CTTTTT
MA0442.1	SOX10	4.80518	0.817776	HDAC6	344	349	-	ATTTGT
MA0442.1	SOX10	4.6688	0.811721	HDAC6	1134	1139	+	CTTTGC
MA0442.1	SOX10	4.6688	0.811721	HDAC6	1576	1581	+	CTTTGA
MA0442.1	SOX10	4.6688	0.811721	HDAC6	80	85	-	CTTTGA
MA0442.1	SOX10	4.6688	0.811721	HDAC6	580	585	-	CTTTGA
MA0442.1	SOX10	4.6688	0.811721	HDAC6	599	604	-	CTTTGC
MA0442.1	SOX10	4.6688	0.811721	HDAC6	1027	1032	-	CTTTGA
MA0442.1	SOX10	4.60516	0.808896	HDAC6	1154	1159	+	CTTGGT
MA0259.1	ARNT::HIF	4.5797	0.802529	HDAC6	1856	1863	+	GGAGGTGG
MA0259.1	ARNT::HIF	4.5661	0.802123	HDAC6	124	131	-	GGACATGG
MA0442.1	SOX10	4.53905	0.805961	HDAC6	1058	1063	+	CAGTCT
MA0442.1	SOX10	4.53905	0.805961	HDAC6	509	514	-	CAGTCT

MA0442.1	SOX10	4.52062	0.805143	HDAC6	32	37	+	AATTGT
MA0442.1	SOX10	4.52062	0.805143	HDAC6	1043	1048	-	AATTGT
